# The *bla*_NDM-1_ and *mcr-1* genes coexist in *Escherichia coli* strain isolated from public trash cans

**DOI:** 10.1093/jacamr/dlae132

**Published:** 2024-08-20

**Authors:** Xiaoqian Long, Jie Li, Hua Yang, Yuehua Gao, Jiangang Ma, Xiaoqun Zeng, Biao Tang

**Affiliations:** Key Laboratory of Systems Health Science of Zhejiang Province, School of Life Science, Hangzhou Institute for Advanced Study, University of Chinese Academy of Sciences, Hangzhou 310024, China; State Key Laboratory for Managing Biotic and Chemical Threats to the Quality and Safety of Agro-Products, College of Food Science and Engineering, Ningbo University, Ningbo 315000, China; College of Life Science, Liaocheng University, Liaocheng 252000, China; State Key Laboratory for Managing Biotic and Chemical Threats to the Quality and Safety of Agro-Products & Institute of Agro-Product Safety and Nutrition, Zhejiang Academy of Agricultural Sciences, Hangzhou, Zhejiang 310021, China; Key Laboratory of Systems Health Science of Zhejiang Province, School of Life Science, Hangzhou Institute for Advanced Study, University of Chinese Academy of Sciences, Hangzhou 310024, China; State Key Laboratory for Managing Biotic and Chemical Threats to the Quality and Safety of Agro-Products & Institute of Agro-Product Safety and Nutrition, Zhejiang Academy of Agricultural Sciences, Hangzhou, Zhejiang 310021, China; Xianghu Laboratory, Hangzhou 311231, Zhejiang, China; State Key Laboratory for Managing Biotic and Chemical Threats to the Quality and Safety of Agro-Products, College of Food Science and Engineering, Ningbo University, Ningbo 315000, China; Key Laboratory of Systems Health Science of Zhejiang Province, School of Life Science, Hangzhou Institute for Advanced Study, University of Chinese Academy of Sciences, Hangzhou 310024, China; State Key Laboratory for Managing Biotic and Chemical Threats to the Quality and Safety of Agro-Products & Institute of Agro-Product Safety and Nutrition, Zhejiang Academy of Agricultural Sciences, Hangzhou, Zhejiang 310021, China

Carbapenem-resistant Enterobacteriaceae are among the most critical priority pathogens that pose a significant threat to human health. Within the Enterobacteriaceae family, *Escherichia coli* is the predominant bacterium. Colistin serves as a crucial ‘last line’ treatment for infections caused by carbapenem-resistant Enterobacteriaceae. But the coexistence of *bla*_NDM-1_ and *mcr-1* in both Enterobacteriaceae and non-Enterobacteriaceae has become widespread on a global scale. The observed phenomenon is particularly noteworthy in *E. coli* isolated from clinical sources,^1^ animal farming^2^ and wastewater.^3^ It is also noteworthy in clinically isolated *Klebsiella pneumoniae*^4^ and *Pseudomonas aeruginosa.*^5^ Furthermore, the majority of reported resistance due to these two genes in these strains is located on plasmids. Research on antimicrobial resistance genes (ARGs) in environmental health is crucial, especially in locations such as public trash cans where human waste is disposed. These sites may contain ARGs that pose risks of transmission to humans, either through airborne dissemination or direct contact with sanitation workers. Given the role of waste disposal in the urban ecosystem, these aspects warrant comprehensive investigation within the ‘One Health’ framework, which emphasizes the interconnectedness of human, animal and environmental health. This holistic approach is essential for understanding and mitigating the potential public health impacts associated with antimicrobial resistance. In this study, we found for the first time that *bla*_NDM-1_ and *mcr-1* genes coexist in *E. coli* isolated from public trash cans and that *mcr-1* is located on a plasmid but *bla*_NDM-1_ is located on the chromosome.

From December 2022 to April 2023, *E. coli* strain ECSD228 carrying *bla*_NDM-1_ and *mcr-1* was isolated from 253 cotton swab samples collected from trash cans in Shandong Province. Concurrently, antimicrobial susceptibility testing was performed using a microbroth dilution method, and the breakpoints for each antimicrobial agent were determined according to CLSI standards. The strain ECSD228 was found to be resistant to both meropenem and colistin, with MIC values of 4 mg/L (Table S1, available as Supplementary data at *JAC-AMR* Online). Illumina NovaSeq 6000 and Oxford Nanopore GridION sequencing platforms were then used, followed by hybrid assembly using Unicycler v0.5.0. The results revealed that strain ECSD228 contained six plasmids, and ResFinder analysis predicted the presence of additional ARGs alongside *bla*_NDM-1_ and *mcr-1* in strain ECSD228 (Table S2). MLST analysis demonstrated that ECSD228 belonged to ST617.

The WGS results revealed that *mcr-1* was located on the 66 460 bp circular plasmid pE228-MCR-66K, with an average GC content of 43%. This plasmid belongs to the IncI2 type, which is known for its high prevalence in carrying the *mcr-1* gene.^6^ The plasmid pE228-MCR-66K not only carries *mcr-1* but also contains an additional ARG called *bla*_CTX-M-55_. Comparative analysis using full plasmid BLAST query demonstrated that pE228-MCR-66K exhibits close similarity with other IncI2 plasmids. Specifically, pE228-MCR-66K shared 99.98% identity with pEC16-50-MCR, pM-199-C35, pMCR-M21015, pSH13G841, pSh487-m4 and pJD053-MCR59K; moreover, their coverage rates exceeded 90%, indicating a high likelihood of horizontal transfer of resistance genes among IncI2 plasmids.^7^ Additionally, pE228-MCR-66K contains the type IV secretion system (T4SS), which is conserved in all IncI2 plasmids and is responsible for plasmid transfer (Figure 1a). Further genetic analysis revealed that *mcr-1* is integrated downstream of the *nikB* gene, and the genetic structure ‘*traL-mobC-nikA-nikB-mcr-1-pap2-traE*’ is highly similar to plasmids pJD053-MCR-59K, pSH15G1531 and pSh069-m6 of the plasmid type IncI2 (Table S3). The *mcr-1-pap2* element has been horizontally transferred into a different plasmid skeleton, again indicating that *mcr-1* has become widespread.^8^ However, compared with the original *mcr-1* carrier plasmid (pHNSHP45) from China, the absence of the IS*Apl1* gene upstream of the *mcr-1* gene increased the stability of *mcr-1* in the plasmid (Figure 1b). Conjugative transfer plays a pivotal role in the spread of plasmid-borne resistance genes, posing a significant threat to public health. Conjugative transfer assays employed azide-resistant *E. coli* J53 as the recipient strain to confirm the transferability of pE228-MCR-66K carrying the *mcr-1* gene. It was observed that despite *mcr-1* being present on the plasmid pE228-MCR-66K, transfer to the recipient strain did not occur in this study. To further validate the potential transfer of the *mcr-1* gene, we conducted natural transformation experiments to assess the ability of DNA fragments carrying the resistance gene to undergo natural transformation into recipient bacteria; however, no positive results were obtained (Figure S1).

**Figure 1.**
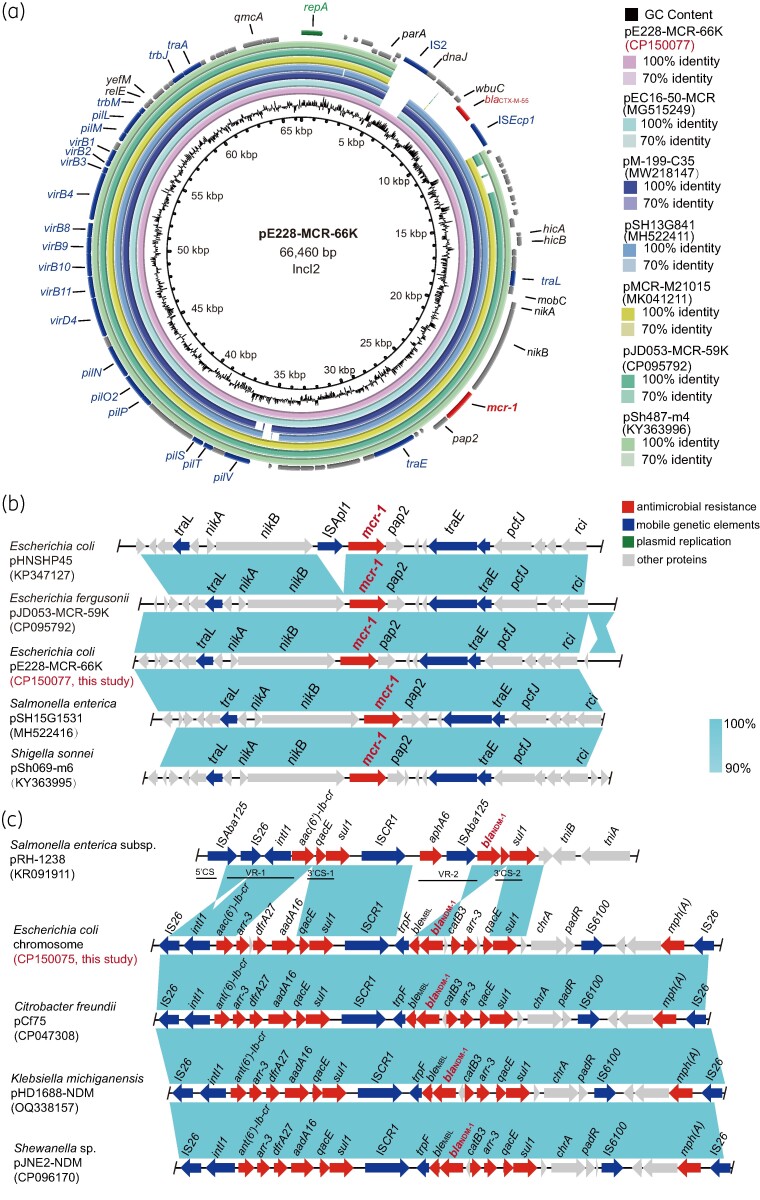
Genetic characteristics of *mcr-1* in *E. coli* ECSD228, and genetic environment of *bla*_NDM-1_ gene. (a) Comparison of plasmids of *mcr-1*-carrying plasmids pE228-MCR-66K, pEC16-50-MCR, pM-199-C35, pMCR-M21015, pSH13G841, pSh487-m4 and pJD053-MCR-59K. (b) The genetic background of *mcr-1* gene compared with the reference plasmids pHNSHP45, pJD053-MCR-59K, pE228-MCR-66K, pSH15G1531 and pSh487-m6. (c) Comparison diagram of *Salmonella enterica* subsp., *E. coli* ECSD228, *Citrobacter freundii* L75, *Klebsiella michiganensis* HD1688 and *Shewanella* sp. JNE2. The red arrows indicate the ARGs, the blue arrows indicate mobile genetic elements, the green arrows indicate plasmid replication and the grey arrows indicate other proteins. VR, variable region; 3′CS, 3′ conserved segment; 5′CS, 5′ conserved segment.

There are a few reports that *bla*_NDM-1_ is located on chromosomes. In addition to *bla*_NDM-1_, the strain ECSD228 also carried other ARGs: *aadA16*, *sul1*, *dfrA27*, *arr-3* and *aac(6′)-Ib-cr* are located in an 11.7 kb multiple antimicrobial resistance region. This region contains a complex class 1 integron, along with a 5′ conserved segment (5′CS), two 3′CSs, a common region IS*CR1*, and two variable regions (VRs), components that in turn carry AMR genes. *bla*_NDM-1_ is embedded in VR-2 attached to the resistance gene *arr-3* and *catB3*, VR-2 and 3′CS-2 linked by IS*CR1*, and can form a modular structure: 5′CS (*intI1*)-VR-1 (*aac (6′)-Ib-cr*, *arr-3*, *dfrA27*, *aadA16*)-3′CS-1 (*qacE-sul1*)-IS*CR1*-VR-2 (*trpF*, *ble*_MBL_, *bla*_NDM-1_, *catB3*, *arr-3*)-3′CS-2 (*qacE-sul1*). In this study, the genetic context (IS*CR1*-*trpF*-*ble*_MBL_-*bla*_NDM-1_-*catB3*-*arr-3*-*qacE-sul1*) of *bla*_NDM-1_ located on chromosomes showed high similarity with pCf75, pHD1688-NDM and pJNE2-NDM belonging to the IncA/C2 plasmid types. However, different from pRH-228, the downstream link of IS*CR1* is not *traF*, but *aphA6* and IS*Aba125*. At the same time, there is deletion of three resistance genes, *catB3*, *qacE* and *arr-3*, between *ble*_MBL_ and *sul1* genes downstream of *bla*_NDM-1_, and the deletion of *arr-3*, *dfrA27* and *aadA16* between *aac(6′)-Ib-cr* and *qacE* genes upstream of *bla*_NDM-1_. This means that the ability of IS*CR1*-mediated class 1 integrons to capture different kinds of resistance genes in different bacteria is not completely consistent.^9^ The *bla*_NDM-1_ gene is primarily transmitted through plasmids, and the presence of insertional elements IS*26,* IS*CR1* and IS*6100* can facilitate the integration of the ARG from the plasmid to the more stable chromosome during transmission.^10^

To the best of our knowledge, this represents the first documented occurrence of both *bla*_NDM-1_ and *mcr-1* in *E. coli* strains isolated from public trash cans, signifying the dissemination of strains carrying *mcr-1* and *bla*_NDM-1_ beyond clinical and animal settings into the public domain, thereby significantly augmenting the risk to public health security. This underscores the imperative for continuous surveillance of polymyxin-resistant and carbapenem-resistant microorganisms by employing a ‘One Health’ approach to mitigate the proliferation of antibiotic-resistant bacteria.

## Supplementary Material

dlae132_Supplementary_Data

## Data Availability

The complete genome sequences of strain ECSD228 were deposited at GenBank with accession numbers CP150075-CP150081.

## Supplementary data

Figure S1 and Tables S1 to S3 are available as Supplementary data at *JAC-AMR* Online.
