# Supplementary material for: The blaNDM-1 and mcr-1 genes coexist in Escherichia coli strain isolated from public trash cans
Source: JAC Antimicrob Resist. 2024 Aug 20;6(4):dlae132. doi: 10.1093/jacamr/dlae132 (PMC11334060; doi:10.1093/jacamr/dlae132)
Supplement: dlae132_Supplementary_Data [file dlae132_supplementary_data.docx]

**
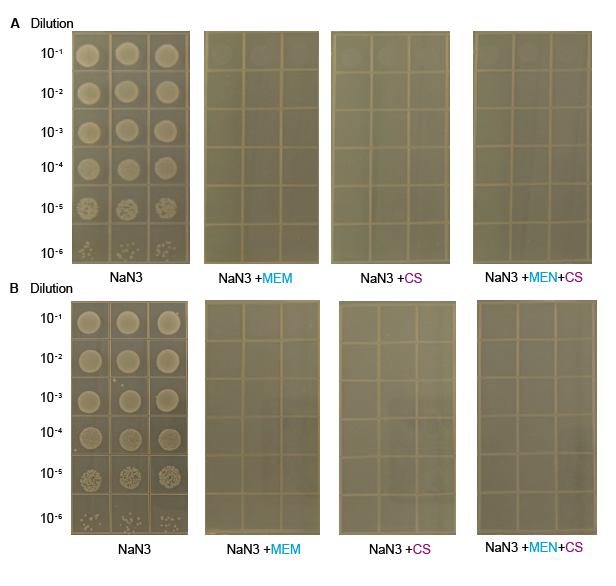
**

**Figure S1** (A) Conjugation transfer test of *E. coli* ECSD228 with sodium azide-resistant *E. coli* J53 served as the recipient strain. (B) Natural transformation test of DNA genome from *E. coli* ECSD228 with sodium azide-resistant *E. coli* J53 served as the recipient strain. From left to right are the screening results under the selective pressure of sodium azide, sodium azide and meropenem, sodium azide and colistin, sodium azide, colistin and meropenem.

**Table S1.** Genomic characteristics of *E. coli* strain ECSD228.

| Chromosome  / plasmid | Accession  numbers | Type | Size (bp) | | GC content (%) | Resistance genes |
| --- | --- | --- | --- | --- | --- | --- |
| chromosome | CP150075 | - | 5, 049,964 | | 51 | *bla*_NDM-1_*, aadA16, aac(6')-Ib-cr, mph(A), erm(B), erm(42), catB3, arr-3, sul1, dfrA27* |
| pE228-137K | CP150076 | IncFII | 137,940 | 52 | | *aac(3)-Iid, aadA22, mph(A), sul2* |
| pE228-MCR-66K | CP150077 | IncI2 | 66,460 | 43 | | *mcr-1, bla*_CTX-M-55_ |
| pE228-40K | CP150078 | IncX1 | 40,935 | 53 | | *aph(6)-Id, aph(3'')-Ib, aph(3')-IIa, aadA2, rmtB，bla*_TEM-1B_*, floR, sul1, sul2，tet*(A)*, dfrA12* |
| pE228-3.1K | CP150079 | - | 3,392 | 53 | | - |
| pE228-3.2K | CP150080 | - | 3,373 | 55 | | - |
| pE228-1.9K | CP150081 | Col (MG828) | 1,906 | 44 | | - |

-, none

**Table S2.** MICs and antimicrobial resistance genes of strain ECSD228.

| **Antibiotics** | **MIC（mg/L）** | **Interpretation** | **ARGs** |
| --- | --- | --- | --- |
| Aampicillin (AMP) | ＞512 | resistant | *bla*_CTX-M-55_ |
| Amoxicillin/clavulanate (A/C) | ＞512/256 | resistant | - |
| Gentamicin (GEM) | ＞512 | resistant | *aph(3')-IIa* |
| Streptomycin (SPT) | ＞512 | resistant | *aadA16, aadA22, aadA2* |
| Tetracycline (TET) | ＞512 | resistant | *tet*(A) |
| Florfenicol (FFC) | ＞512 | resistant | - |
| Sulfisoxazole (SF) | ＞512 | resistant | *sul1, sul2* |
| Trimethoprim/sulfamethoxazole (SXT) | 32/608 | resistant | - |
| Ceftiofur (CEF) | ＞256 | resistant | - |
| Ceftazidime (CAZ) | ＞256 | resistant | - |
| Enrofloxacin (ENR) | ＞32 | resistant | - |
| Ofloxacinn (OFL) | 64 | resistant | - |
| Meropenem (MEM) | 4 | resistant | *bla*_NDM-1_ |
| Colistin (CS) | 4 | resistant | *mcr-1* |

-, none

**Table S3.** Comparison of plasmid pECSD228-MCR-66K in this study with other homologous plasmids and relative strains information.

| **plasmids** | **plasmid type** | **sequence coverage** | **nucleotide identity** | **accession number** | **strain** | **species** |
| --- | --- | --- | --- | --- | --- | --- |
| pJD053-MCR-59K | IncI2 | 100% | 99.96% | CP095792 | EF21JD053 | *Escherichia fergusonii* |
| pSH15G1531 | IncI2 | 100% | 99.97% | MH522416 | SH15G1531 | *Salmonella enterica* |
| pSh069-m6 | IncI2 | 100% | 99.97% | KY363995 | SH13Sh069 | *Shigella sonnei* |
